# Antibody production and characterization of the nucleoprotein of sever fever with thrombocytopenia syndrome virus (SFTSV) for effective diagnosis of SFTSV

**DOI:** 10.1186/s12985-023-02173-1

**Published:** 2023-09-07

**Authors:** Kyungha Lee, Min Ji Choi, Man-Ho Cho, Dong Ok Choi, Seong-Hee Bhoo

**Affiliations:** 1 Graduate School of Biotechnology, Kyung Hee University, Yongin, 17104 Korea; 2 Department of Genetics and Biotechnology, Kyung Hee University, Yongin, 17104 Korea; 3 Bore Da Biotech, Seongnam-si, Gyeonggi-do 13209 Korea; 4 Graduate School of Green-Bio Science, Kyung Hee University, Yongin, 17104 Korea

**Keywords:** Severe fever with thrombocytopenia syndrome virus, *Dabie bandavirus*, Nucleoprotein, Diagnosis

## Abstract

**Background:**

Severe fever with thrombocytopenia syndrome (SFTS) is an infectious disease caused by the *Dabie bandavirus*, [or SFTS virus (SFTSV)] that has become increasingly widespread since it was first reported in 2009. The SFTSV comprises three essential single-stranded RNA gene segments, with the S segment encoding the nucleocapsid (N) protein. Since the N protein is the most abundant and stable viral protein, it is a useful diagnostic marker of infection. Various SFTSV N-protein-based detection methods have been developed. However, given the limited research on antibodies of an SFTSV N-protein, here we report the characterization of the antibodies against SFTSV N protein especially their mapping results which is essential for more efficient and optimized detection of SFTSV.

**Methods:**

To generate SFTSV-N-protein-specific monoclonal antibodies, recombinant full-length SFTSV N protein was expressed in *E. coli*, and the purified N protein was immunized to mice. The binding epitope positions of the antibodies generated were identified through binding-domain mapping. An antibody pair test using a lateral flow immunoassay (LFIA) was performed to identify effective diagnostic combinations of paired antibodies.

**Results:**

Nine monoclonal antibodies specific for the SFTSV N protein were generated. Antibodies #3(B4E2) and #5(B4D9) were specific for sequential epitopes, while the remainder were specific for conformational epitopes. Antibody #4(C2G1) showed the highest affinity for the SFTSV N protein. The binding domain mapping results indicated the binding regions of the antibodies were divided into three groups. The antibody pair test demonstrated that #3(B4E2)/#4(C2G1) and #4(C2G1)/#5(B4D9) were effective antibody pairs for SFTSV diagnosis.

**Conclusions:**

Effective virus detection requires at least two strong antibodies recognizing separate epitope binding sites of the virus antigen. Here, we generated SFTSV-N-protein-specific monoclonal antibodies and subsequently performed epitope mapping and an antibody pair test to enhance the diagnostic efficiency and accuracy of SFTSV. Confirmation of epitope mappings and their combination immune response to the N protein provide valuable information for effective detection of SFTSV as well as can respond actively to detect a variant SFTSV.

**Supplementary Information:**

The online version contains supplementary material available at 10.1186/s12985-023-02173-1.

## Background

Severe fever with thrombocytopenia syndrome (SFTS) is an infectious tick-borne disease caused by the SFTS Virus (SFTSV), which emerged in China in 2009 [1]. Cases present with various clinical signs and symptoms, including fever, thrombocytopenia, leukocytopenia, and gastrointestinal symptoms [2, 3]. Since the first cases report, SFTS has been reported in most East Asian countries, including Korea and Japan [4, 5], with increasing frequency [6–8]. In Korea, SFTS was classified as a national legal infectious disease in 2020, and has since been closely monitored [9]. As such, the rapid and efficient diagnosis of SFTSV has become a national concern along with the development of vaccines and therapeutics.

Initially, SFTSV was classified within the genus Phlebovirus of the family Phenuiviridae, but it was later reclassified into the genus Bandavirus and renamed the *Dabie bandavirus* based on the 2020 ICTV (International Committee on Taxonomy of Viruses) taxonomy release [10, 11]. Regardless, the name SFTSV, derived from the symptoms, is still commonly used among scientists. Similar to viruses in the Phenuiviridae family, the SFTSV is a negative-strand RNA virus that possesses three single-stranded RNA segments: large (L), medium (M), and small (S) [12, 13]. The L segment encodes an RNA-dependent RNA polymerase (RdRp); the M segment encodes a glycoprotein (Gn,Gc); and the S segment encodes two proteins, a nucleocapsid protein (N) and a non-structural protein (NS).

The N protein is important for virus replication and transcription because it protects the viral RNA through encapsidation [14–17]. The N protein binds with the viral RNA and forms ring-like oligomers that facilitate packing and stabilization of the viral RNA. Furthermore, the N protein plays a critical role in virion assembly by forming a ribonucleoprotein (RNP) with the L protein (RNA polymerase) [14]. The N protein is the most abundant and highly conserved protein in most viruses [18–20]. Therefore, this protein is often targeted in viral detection assays [21–23]. Various N-protein-based SFTSV detection methods have been developed [24–26]; however, the known methods provide limited information about their antibodies and binding epitopes.

In this study, we postulated that immune response to the SFTSV N protein is limited due to the small size of the SFTSV-N-protein oligomer in native conditions. Therefore, we produced SFTSV-N-protein-specific antibodies to assess the immune response to SFTSV N protein, the antibody binding affinity, and the epitopes involved. We conducted an antibody pair test using the generated antibodies to find the best pairs for SFTSV detection. The results provided important information for future development of more effective methods to diagnose SFTS caused by SFTSV, such as a commercial rapid antigen detection test kit.

## Materials and methods

### Expression and purification of recombinant full-length and truncated SFTSV N protein

The recombinant SFTSV N protein was cloned into a pET21(a) vector with N-terminal His-tag and expressed in the Rosetta™ 2 (DE3; BL21 derivative) cell line. The transformed cells were induced with IPTG (Isopropyl-ß-D-thio-galactopyranoside) and lysed by sonication in binding buffer (100 mM Tris–HCl, 200 mM NaCl, 10 mM Imidazole, pH 8.0). The total soluble protein was purified using Ni–NTA agarose (Qiagen, Valencia, CA) according to the manufacturer’s recommendations.

Truncated SFTSV N proteins were designed to remove the predicted α–helix from the N-terminus (Additional file 1: Fig. S1) [27–29]. Five N proteins truncated at the N-terminus, dN1(13–256 AA), dN2(33–245 AA), dN3(47–245 AA), dN4(65–245 AA), and dN5(75–245 AA), were cloned into pET21(a) with an N- or C- terminal His-tag for purification and expressed in BL21 or Rosetta cells. The purification procedure was performed as described above. The truncated dN1, dN4, and dN5 proteins were highly soluble, but dN2 and dN3 required solubilization in 0.8 M urea.

### SDS-PAGE and native-PAGE

Purified protein samples were separated on 12% polyacrylamide gels and stained using Coomassie brilliant blue R-250 (Sigma-Aldrich, USA). For the native-PAGE, 4–15% polyacrylamide gels and running buffer without SDS were used, and gel runs were performed in ice water.

### Western blot analyses

Protein samples were separated by SDS-PAGE and transferred to PVDF (polyvinylidene difluoride) membranes (Invitrogen, USA) for western blotting. An anti-His-tag antibody (Santa Cruz, USA) and the SFTSV-N- protein-specific monoclonal antibodies were used to confirm the immune response to the injected recombinant SFTSV N protein antigen. These primary antibodies were used at 5,000-fold dilution with 3% (w/v) skim milk in PBS. The horseradish-peroxidase (HRP)-conjugated anti-mouse IgG secondary antibody was diluted 10,000-fold with 3% skim milk in PBS. The signals were detected using ECL (Enhanced Chemiluminescence) (GE Healthcare, USA).

### Monoclonal antibody production

After mixing the full-length SFTSV N protein (1 mg/mL) with an equal volume of incomplete adjuvant (Sigma, USA), 150 µL of the mixture was injected into each footpad of BALB/c mouse three times at three-week intervals. After immunization, the lymph nodes were collected to obtain B cells that then were fused with Sp2/O myeloma cells to produce hybridoma cells [30]. The hybridoma cells were incubated for two weeks in a 96-well plate. Cell culture medium containing the antibodies was transferred into a new 96-well plate coated with 1 µg/mL of SFTSV N protein to test their immune responses using an indirect ELISA method. The cells confirmed to produce SFTSV N protein-specific antibodies were processed by serial dilution, transferred to a new 96-well plate, and incubated for one week. The ELISA test was performed again to confirm antibody production by the hybridoma cells. The verified cells were then cultured up to a 250 mL volume through sequential scale-up and were concentrated as appropriate. About 0.5 mL of the antibody-producing hybridoma cells (1 × 10^6^) were injected into a BALB/c mouse intraperitoneally, and monoclonal antibodies were harvested and purified from ascites fluid.

### Dot blot analysis

Dot blot analysis were conducted to confirm that SFTSV monoclonal antibodies were specific for the native conformation and epitopes of SFTSV N protein. Expressed and purified full-length and truncated SFTSV N protein samples were loaded onto a nitrocellulose membrane. After blocking, each anti-SFTSV primary antibody was applied at a 5000-fold dilution with 3% (w/v) skim milk in PBS. The secondary antibody and detection process were the same as for the western blot procedure.

### Indirect ELISA

Recombinant full-length SFTSV N protein (3 µg/mL) was added to 96-well ELISA plates overnight at 4 °C. The plates were washed with PBS-T (0.05%, v/v) and blocked with 3% (w/v) skim milk in PBS for 2 h. After washing out the blocking solution, each SFTSV monoclonal antibody in PBS was added according to a concentration gradient. Anti-His6 antibody and anti-2B8 antibody (Biojane Co., Ltd., South Korea) were used as the positive and negative controls, respectively, for experimental validation. After a 1 h incubation, the plates were washed with PBS-T, HRP-conjugated goat anti-mouse IgG diluted 1:10,000 in PBS was added, and the plates were incubated for 1 h. After washing, TMB (3,3′,5,5′-tetramethylbenzidine) substrate was added for reaction with HRP until it was stopped by addition of sulfuric acid. The optical density of the plate was read at 450 nm using a microplate reader (Thermofisher, USA).

### Antibody pair test for diagnosis

A paired-antibody test was performed using the lateral flow immunoassay (LFIA) method. Half-strip testing was carried out in advance using all antibody combinations [31]. Half-strip testing was performed without a sample and conjugate pad. The test line was coated with each SFTSV antibody as a capture antibody (2 mg/mL), and the control line was coated with goat anti-mouse IgG (2 mg/mL). The assay was performed using 5 µL of each SFTSV antibody conjugated with gold as a detection antibody and mixed with 45 µL of SFTSV N protein (100 ng/mL) or 45 µL of 0.1 M PBS as a negative control (1% Tween-20, pH 7.4). Each strip was dipped in the mixed solution for absorption. After 12 min, the visibility of the red lines on the strips was compared among samples, and their immune responses were analyzed to select good pairs. Selected antibody pairs were made into complete test kits. The full strip had a sample pad and a conjugate pad that held the gold-conjugated SFTSV antibody. The test and control lines of the strip were the same as those of the half strip test. For each pair, three identical kits were prepared and 100 µL of 0.1 M PBS, 1 ng/mL of SFTSV N protein, and 0.1 ng/mL of SFTSV N protein were applied, respectively. After waiting for 12 min, the visibility of the lines on the kits was analyzed.

### Sequence alignment and secondary structure depiction

The N protein sequences of three *Bandavirus* species were collected from GeneBank; SFTSV (*Dabie bandavirus*) (GenBank: KC505125.1), *Guertu bandavirus* (GenBank: QBQ64952.1), and *Heartland bandavirus* (GenBank: AFP33391.1). ESPript (espript.ibcp.fr) software was used for sequence alignment and secondary structure depiction. Secondary structure information files for the SFTSV pentamer (4J4U) or hexamer (4J4R) were input from PDB for reference (Additional file 1: Fig. S1) [27–29].

### Denaturation test

Since truncated dN2 and dN3 required solubilization in 0.8 M urea solution, we assessed whether or not their native structures were maintained. Full-length and truncated dN2 and dN3 SFTSV N proteins were subjected to four denaturation factors; urea (0.8 M, 2 M, 4 M, or 6 M), SDS, β-mercaptoethanol, or heat.

## Results

### Expression of the recombinant SFTSV N protein and production of monoclonal antibodies

**Fig. 1:**
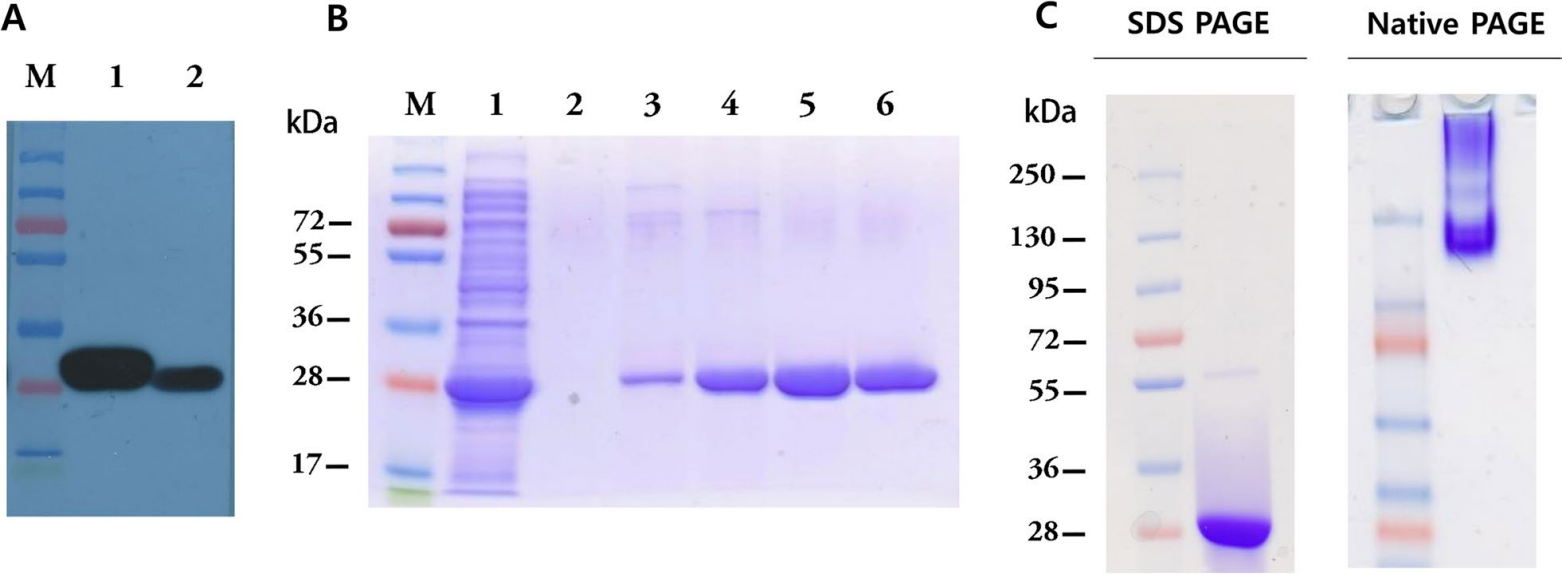
Expression and purification of SFTSV N protein. **A** Western blot analyses of full-length denatured SFTSV N protein. Anti-his-tag antibody was used as the primary antibody. Lane M—molecular size marker; Lane 1—His-GFP as the positive control for His antibody; Lane 2—expressed SFTSV N protein. **B** SDS-PAGE analysis of purified SFTSV N protein. Lane M = molecular size marker; Lane 1 = non-bound (flow through); Lane 2 = washed with binding buffer (100 mM Tris, 200 mM NaCl, 10 mM imidazole, pH8.0), Lanes 3–6: Purified SFTSV N protein eluted with 50 mM, 100 mM, 150 mM, or 200 mM imidazole, respectively. **C** Comparison of native and denatured SFTSV N protein after separation by SDS-PAGE or native-PAGE. The native-PAGE results indicate that the native SFTSV N protein was oligomerized

The recombinant SFTSV N protein with the N-terminal His-tag was expressed and purified. Expression and purification were confirmed by western blot analyses with an anti-His-tag antibody (Fig. 1A, B). The results showed that the purified recombinant SFTSV N protein was highly pure and of the correct size (28 kDa). In addition, native PAGE analysis of the purified non-denatured N protein showed the protein to be present as oligomers [27] as expected (Fig. 1C).

**Fig. 2:**
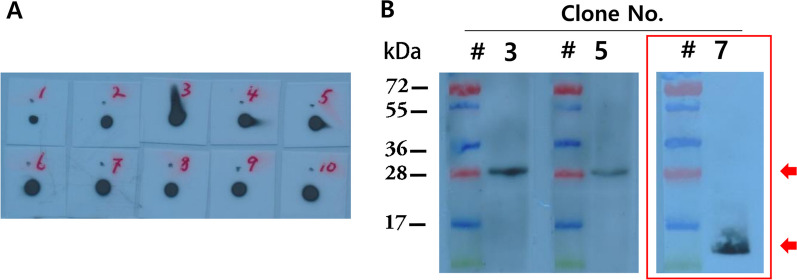
Identification of specific epitopes on SFTSV N protein and type of antibody produced in response to SFTSV N protein immunization. **A** Dot blot analysis of full-length native SFTSV N protein using each anti-SFTSV-N-protein antibody produced in response to immunization of mice. All antibodies detected the native SFTSV N protein. **B** Western blot analysis of full-length denatured SFTSV N protein using each anti-SFTSV-N-protein antibody produced in response to immunization of mice. Clones #3(B4E2) and #5(B4D9) detected the denatured SFTSV N protein of correct size (28 kDa), and clone #7(C4C12) detected an unexpected protein. Data for other antibodies for which no bands appeared are not shown

Purified SFTSV N protein was injected into mice to generate target-protein-specific antibodies as discussed in the methods. Ten monoclonal antibodies recognizing the SFTSV N protein were obtained through the ELISA test (data not shown). Dot-blot analyses were carried out to confirm that the antibodies generated were specific for the SFTSV N protein under non-denaturing native conditions (Fig. 2A). As expected from the initial ELISA screening results, all antibodies showed a positive immune signal, suggesting that all screened antibodies were specific for the native SFTSV N protein.

Western blot analyses were carried out to identify the characteristics of antibody generated (Fig. 2B). Antibody #7(C4C12) showed a western blot signal at a different size from that of the SFTSV N protein, indicating that antibody clone #7 was not specific for the SFTSV N protein. Of the remaining nine antibodies, only clones #3(B4E2) and #5(B4D9) produced bands at the size corresponding to the denatured protein on western blots. Therefore, we concluded that antibodies #3(B4E2) and #5(B4D9) are sequential antibodies that recognize the sequential epitopes of SFTSV N protein. The other seven were considered conformational antibodies that recognized conformational epitopes of the target protein. The results are summarized in Table1.

**Table 1:** Summary of anti-SFTSV-N-protein antibodies produced in response to administration of SFTSV N protein to mice

SFTSV Ab clones		Active against SFTSV N protein antigen		Antibody type
Clone #	Clone name	Dot blot native proteins	Western blot denatured proteins	Sequential/conformational
1	3H6	Positive	Negative	Conformational
2	1A6	Positive	Negative	Conformational
3	B4E2	Positive	Positive	Sequential
4	C2G1	Positive	Negative	Conformational
5	B4D9	Positive	Positive	Sequential
6	A2H12	Positive	Negative	Conformational
7	C4C12	Positive	Negative	Non-specific for SFTSV
8	B1G12	Positive	Negative	Conformational
9	4G1	Positive	Negative	Conformational
10	B2H12	Positive	Negative	Conformational

### Indirect ELISA

**Fig. 3:**
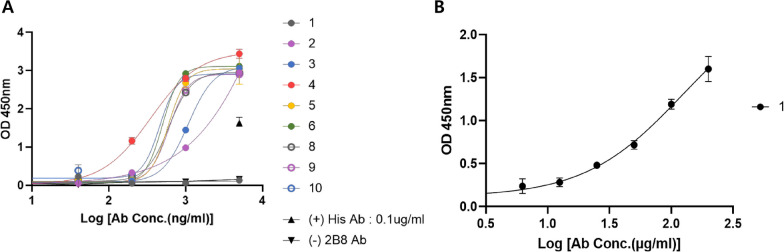
Indirect ELISA of produced SFTSV monoclonal antibodies. Anti-SFTSV-N antibodies isolated from immunized mice were used as primary antibodies in indirect ELISA. Purified full-length SFTSV N protein was used as the antigen. **A** Indirect ELISA using nine SFTSV monoclonal antibodies. Five-fold serial dilutions of 5 µg/mL stock solutions of each antibody were used in the assay. Anti-His6 antibody (▲) and 2B8 antibody (▼) were used as the positive and negative controls, respectively. Anti-His6 antibody applied single concentration (0.1 µg/ml) was indicated together with 5 µg/ml (3.7 as log value) in the figure. **B** Indirect ELISA of SFTSV antibody clone #1(3H6). Two-fold dilutions of a 200 µg/mL stock solution of 3H6 were used in the assay

An indirect ELISA was performed to compare the binding of the nine SFTSV antibodies to that of the SFTSV N protein. Seven five-fold serial dilutions of 5 µg/mL stock solutions of each antibody were performed, and the resulting dilutions were incubated in ELISA plate wells coated with 300 ng of recombinant SFTSV N protein. In addition to the SFTSV antibodies, an anti-His6 antibody that recognizes His6-tagged recombinant antigens was employed as a positive control (0.1 µg/mL). Most antibodies showed signals at detection ranges from 0.2 to 1 µg/mL (2.3 to 3 as log value) and signals were saturated at higher concentrations. Notably, antibody #4(C2G1) exhibited the lowest EC_50_ value [32, 33], indicating that it had greater affinity for the SFTSV N protein antigen than the other antibodies (Fig. 3A). However, antibody #1(3H6) showed no perceivable signal at a 5 µg/mL concentration (3.7 as log value), suggesting a lower affinity for the antigen. To confirm these findings, the ELISA was repeated using two-fold serial dilutions of 200 µg/mL stock solutions of antibody. The results indicate that antibody #1(3H6) requires a much higher concentration to bind the antigen and, therefore, had a relatively lower affinity for the antigen (Fig. 3B).

### Production of truncated SFTSV N protein

**Fig. 4:**
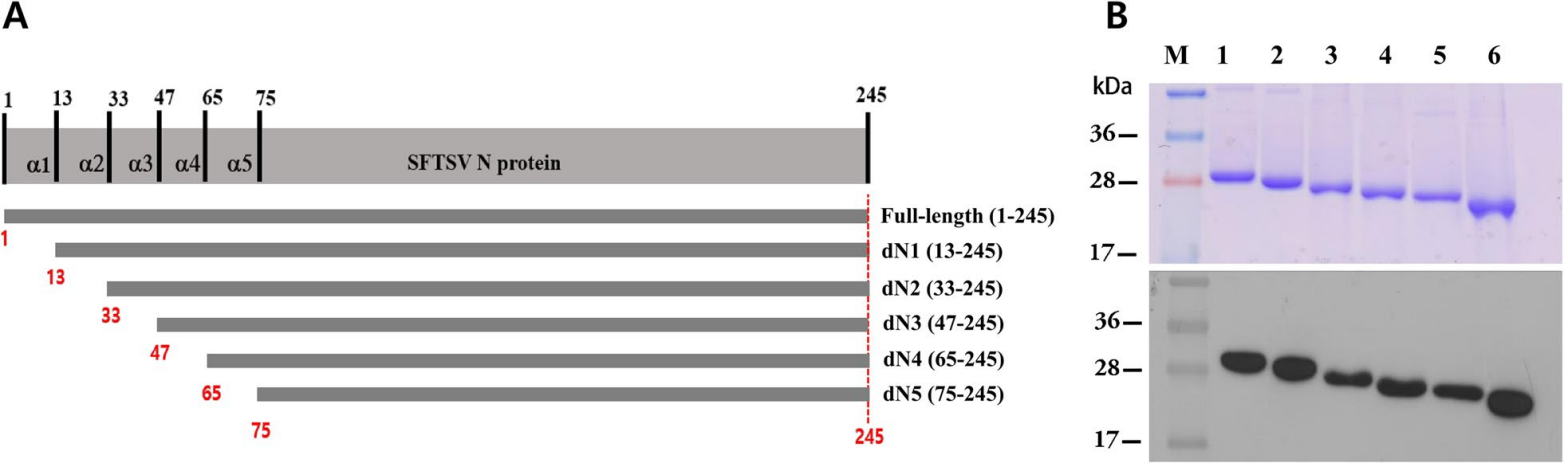
Expression and purification of truncated SFTSV N protein. **A** Scheme of SFTSV N truncation. **B** SDS-PAGE (top) and western blot (bottom) analyses of SFTSV truncates. Anti-his-tag antibody was used as primary antibody in western blot. Lane M—molecular weight marker; Lane 1—full-length SFTSV N protein (1–245); Lanes 2–6—Truncated SFTSV N proteins, dN1 (13–245), dN2 (33–245), dN3 (47–245), dN4 (65–245), and dN5 (75–245), respectively; dN2 and dN3 were lysed and purified in 0.8 M urea

To map the epitopes of SFTSV N protein, we produced serial truncated proteins. The secondary structure of the protein was predicted using ESpript (espript.ibcp.fr) software (Additional file 1: Fig. S1). Based on the predicted secondary structures, cleavage sites within the SFTSV N protein were determined such that the α-helix at the N-terminus could be sequentially removed, and the resulting truncated products were named dN# (Fig. 4A). Five truncated SFTSV N proteins named dN1(13–245), dN2(33–245), dN3(47–245), dN4(65–245, and dN5(75–245) were co-expressed in *E. coli* with His-tag for purification. The SDS-PAGE and western blot results indicated that all truncated SFTSV N proteins were well expressed, of the correct sizes, and highly purified (Fig. 4B).

### Epitope mapping of SFTSV N protein antibodies

**Fig. 5:**
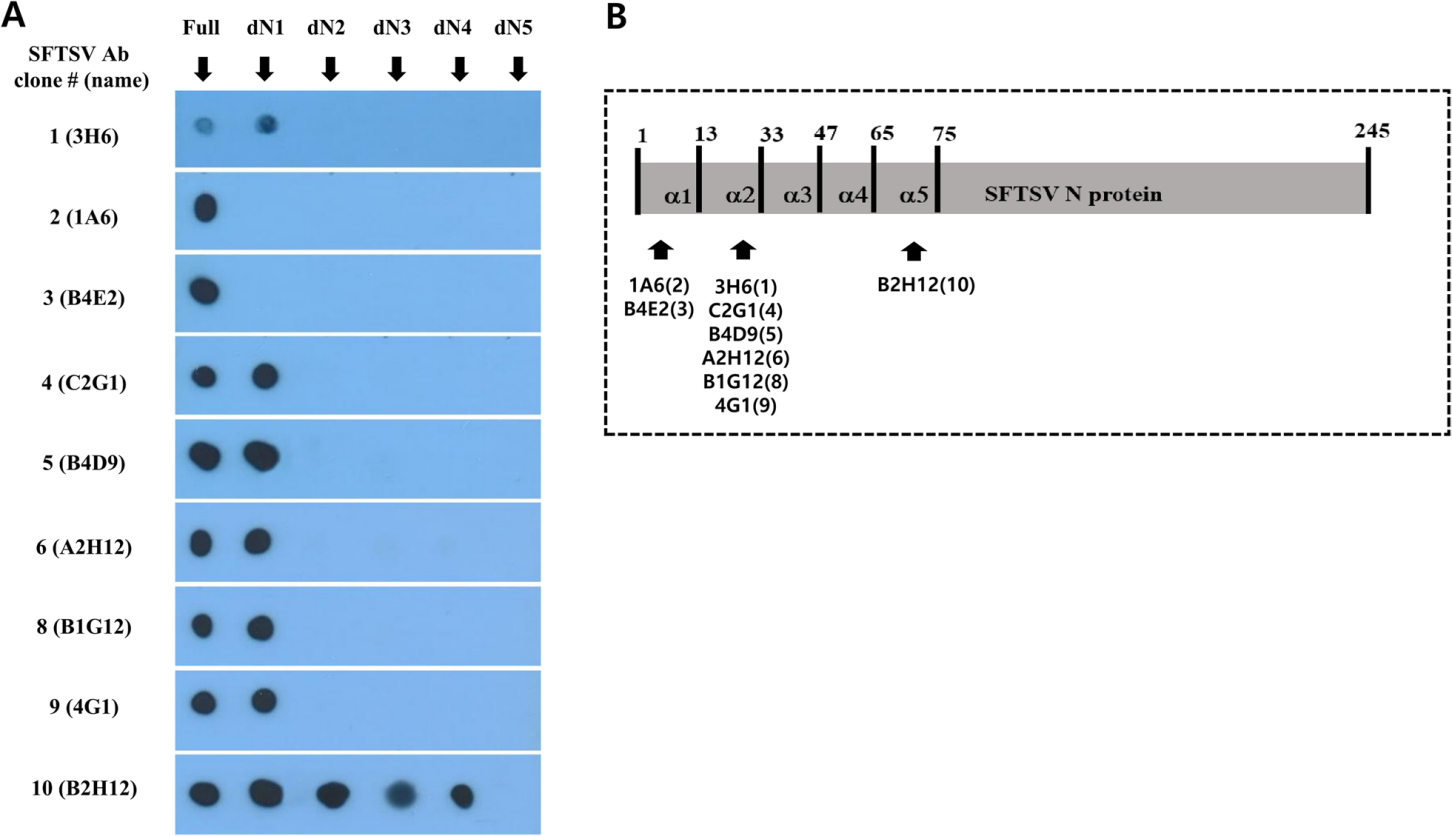
Epitope mapping of SFTSV N protein antibodies. **A** Dot-blot analyses of full-length and truncated SFTSV N proteins. **B** The locations of the epitopes on the SFTSV N protein to which antibodies bound

Epitope mapping was performed to find the binding epitope positions for the nine SFTSV monoclonal antibodies. None of the antibodies, except #10(B2H12), bound to dN2(33–245) through dN5(75–245) truncated SFTSV N proteins (Fig. 5A), suggesting that these eight antibodies bind to the epitopes of the α-helix 1 or 2 section located in the SFTSV N-arm region [17, 27, 34]. Specifically, antibodies #2(1A6) and #3(B4E2) were shown to bind the first α-helix of the N-arm, while antibodies #1(3H6), #4(C2G1), #5(B4D9), #6(A2H12), #8(B1G12), and #9(4G1) bound the second helix of the N arm (α-helix 2). Only antibody #10(B2H12) showed binding to the α-helix 5 section, which is not within the N arm region. The nine SFTSV-N-protein-specific antibodies were categorized into three groups according to epitopes bound: Group 1 (#2,3), Group 2 (#1,4,5,6,8,9), and Group 3 (#10) (Fig. 5B). Truncated dN2 and dN3 proteins were not sufficiently soluble in the typical extraction buffer; for our study, we solubilized these two proteins in 0.8 M urea for purification. Denaturation tests showed the epitope conformations of dN2 and dN3 to be maintained when solubilized in urea (Additional file 1: Fig. S2).

### Antibody pair test

**Fig. 6:**
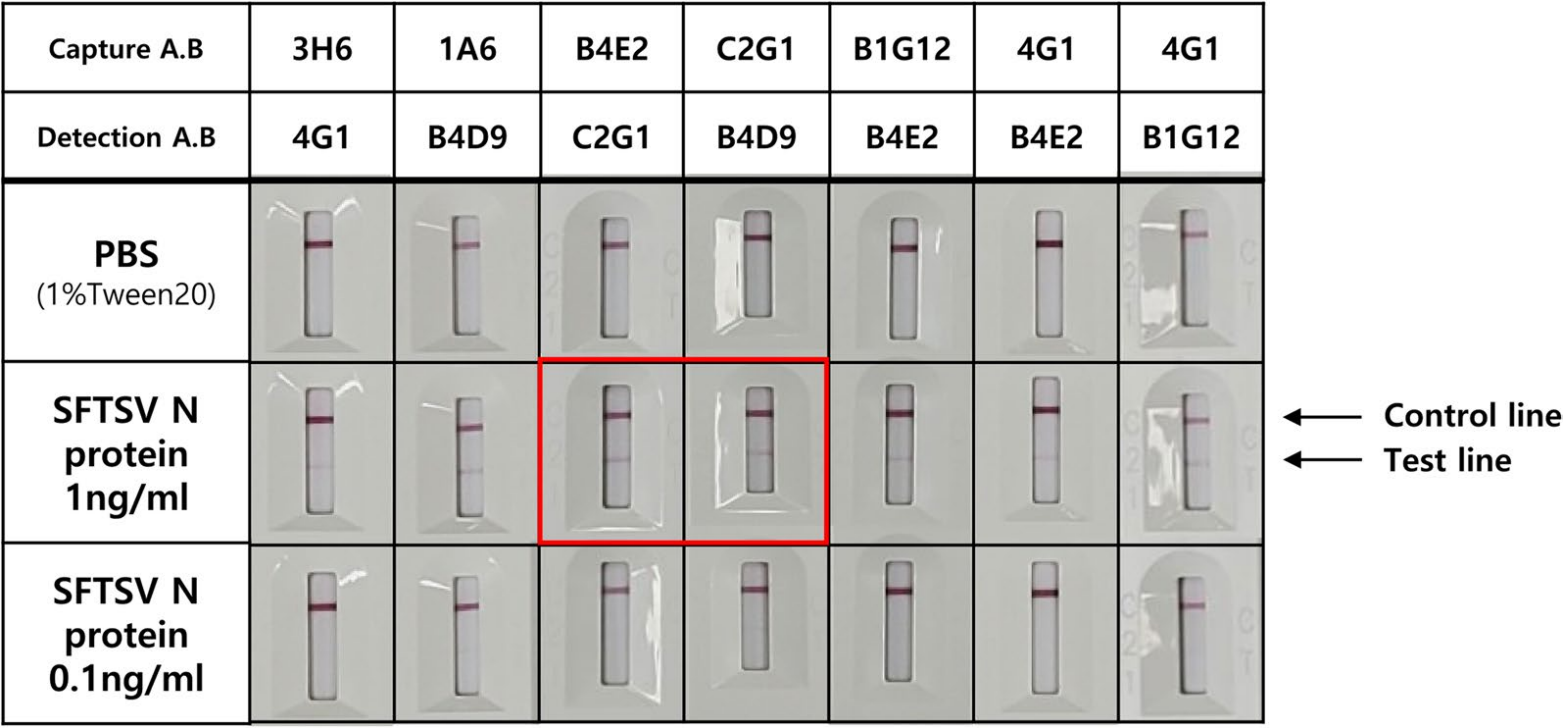
Pair test for SFTSV diagnosis. Diagnostic kits containing the antibody combinations that showed a relatively obvious line in the half test were prepared. Tests were conducted using varying concentrations of full-length SFTSV N protein as the antigen

To select the best combination of antibodies for detection of SFTSV N protein, a lateral flow immunoassay (LFIA) was carried out. A half-strip test was conducted first to verify the functionality of the experimental system (Additional file 1: Fig. S3). Among all possible antibody combinations, seven pairs with relatively clear bands on the SFTSV N protein test line were selected as candidates and processed into complete rapid test kits (Fig. 6).

Two concentrations (1 ng/mL and 0.1 ng/mL) were tested to determine the appropriate antigen concentration. The 1 ng/mL concentration resulted in a faint but visible band on the test line, while the 0.1 ng/ml concentration showed no visible results. Among the antibody pairs tested, the #3(B4E2)-#4(C2G1) and #4(C2G1)-#5(B4E2) pairs produced relatively noticeable lines (red box), suggesting that antibody pairs containing #4(C2G1) had the greatest affinity for the SFTSV N protein (Fig. 3A) and were considered optimal for effective detection of the SFTSV N protein. Furthermore, antibodies #3(B4E2) and #5(B4D9), which were the only sequential antibodies among those tested, were also confirmed to be appropriate partners for development of SFTSV diagnostic tools.

## Discussion

In this study, recombinant SFTSV N protein was expressed in *E. coli*. Mice were immunized with this protein to produce monoclonal antibodies specific for the SFTSV N protein. Two sequential antibodies and seven conformational antibodies were generated. These antibodies showed immune responses to the native SFTSV N protein in dot-blot analyses, and their binding affinities were confirmed through indirect ELISA. In addition, the binding positions of the generated antibodies within the SFTSV N protein were identified. The binding-domain mapping with truncated SFTSV N proteins indicated that the epitopes bound by eight of the nine tested antibodies were localized to narrow α-helix 1 and 2 regions corresponding to the SFTSV N-arm [17, 27, 34]. The N-arm of the SFTSV N protein stretches between adjacent molecules within N protein oligomers. Our study confirmed that the N-arm of antigen, which is relatively exposed on the surface, triggered immune responses in host cells. On the other hand, α-helix 5 is located adjacent to the neighboring N protein molecules in a ring-shaped, making this helix is less exposed than α-helix 1 and 2 [27–29]. However, through the binding of antibody #10(B2H12), it is confirmed that the epitope is at least exposed.

The nine antibodies were classified into three groups according to binding positions. Unexpectedly, the binding sites of six antibodies in Group 2 were localized to a narrow area comprising only the 20 amino acids between positions 13 through 33. Since it is thought that most epitopes that generate immune responses are about 15–30 amino acids in length [35], more detailed epitope mapping is required to determine whether the epitopes bound by these six antibodies overlap; however, detailed mapping was beyond the scope of the current study. For our study, we hypothesized that two antibodies that bind different epitopes would be required to develop an effective SFTSV-antigen-detection tool for diagnostic use. Therefore, we conducted pairwise testing of the nine antibodies using the full-length SFTSV N protein as the target to find the optimal capture-detection antibody combination/s for SFTSV detection. The unexpected result was that positive detection signals were (also) evident for antibody pairs recognizing the same binding epitope position. This finding may be explained by the oligomerization of SFTSV N proteins. The SFTSV N protein is a homo-oligomeric protein (a pentamer or hexamer) that forms a ring-like structure. Therefore, five or six identical epitopes may be present on the oligomer complex molecule [36–38], and captured oligomer complexes of SFTSV N protein by surface-immobilized antibody may leave exposed the remaining epitopes on the other subunits of SFTSV N proteins. Oligomerization of the SFTSV N proteins used in this study was confirmed in the native-PAGE assay (Fig. 1C).

Consequently, the results indicated that the best candidates for the capture-detection pair are antibodies #3, #4, and #5, and that the #3(B4E2)-#4(C2G1) and #4(C2G1)-#5(B4D9) combinations were suitable for use in diagnostic tools. Among the three candidates, antibodies #3(B4E2) and #5(B4D9) were sequential, suggesting that sequential antibodies may be advantageous for a diagnosis system because they detect both native and denatured antigens. Among the two best antibody pairs identified, those of the #3(B4E2)-#4(C2G1) pair recognize different epitopes, while the antibodies of the #4(C2G1)-#5(B4D9) pair recognize the same epitope; however, we do not know whether these two antibodies recognize the exact same epitope. If the exact same epitope is bound by the two antibodies, the results of the pair test can be explained by binding to different N subunits. Considered together, the results indicate that the best pair of antibodies for development of SFTSV diagnostic tools is likely the [#3(B4E2)-#4(C2G1)] pair.

## Conclusions

Given the steady increase in SFTSV infections, development of a rapid and efficient diagnostic tools is of national and global interest. An approach to understanding the SFTSV N protein and its specific antibodies is crucial for optimized diagnosis. We produced SFTSV-N-protein-specific monoclonal antibodies and characterized their biochemical and immunological functions with respect to the SFTSV N protein. Based on the epitope mapping results, we confirmed that most of the anti-SFTSV antibodies generated in immunized mice recognized highly exposed regions of the N protein. In addition, we were able to identify the #3(B4E2)-#4(C2G1) antibody pair as the best for SFTSV detection. The results from this study will serve as crucial insights into detection mutations in the SFTSV N protein and furthermore, these insights will be instrumental for the development of effective commercial diagnostic tools for SFTSV.

### Supplementary Information

**Additional file 1**. Sequence and epitope characteristic analysis of the SFTSV N protein, and pair test of generated antibodies.

## Data Availability

All data generated or analyzed during this study are included in this published article and its supplementary information files. The sequence data of the SFTSV N protein can be accessed through GenBank with ID KC505125.1, the link is https://www.ncbi.nlm.nih.gov/nuccore/KC505125.1

## Abbreviations

SFTS: Severe fever with thrombocytopenia syndrome
SFTSV: SFTS virus
N: Nucleocapsid protein
dN: Deleted N protein
ELISA: Enzyme-linked immunosorbent assay
LFIA: Lateral flow immunoassay
