# Supplementary material for: Antibody production and characterization of the nucleoprotein of sever fever with thrombocytopenia syndrome virus (SFTSV) for effective diagnosis of SFTSV
Source: Virol J. 2023 Sep 7;20:206. doi: 10.1186/s12985-023-02173-1 (PMC10486111; doi:10.1186/s12985-023-02173-1)
Supplement: Supplementary file 1 — Additional file 1. Sequence and epitope characteristic analysis of the SFTSV N protein, and pair test of generated antibodies. [file 12985_2023_2173_MOESM1_ESM.pdf]

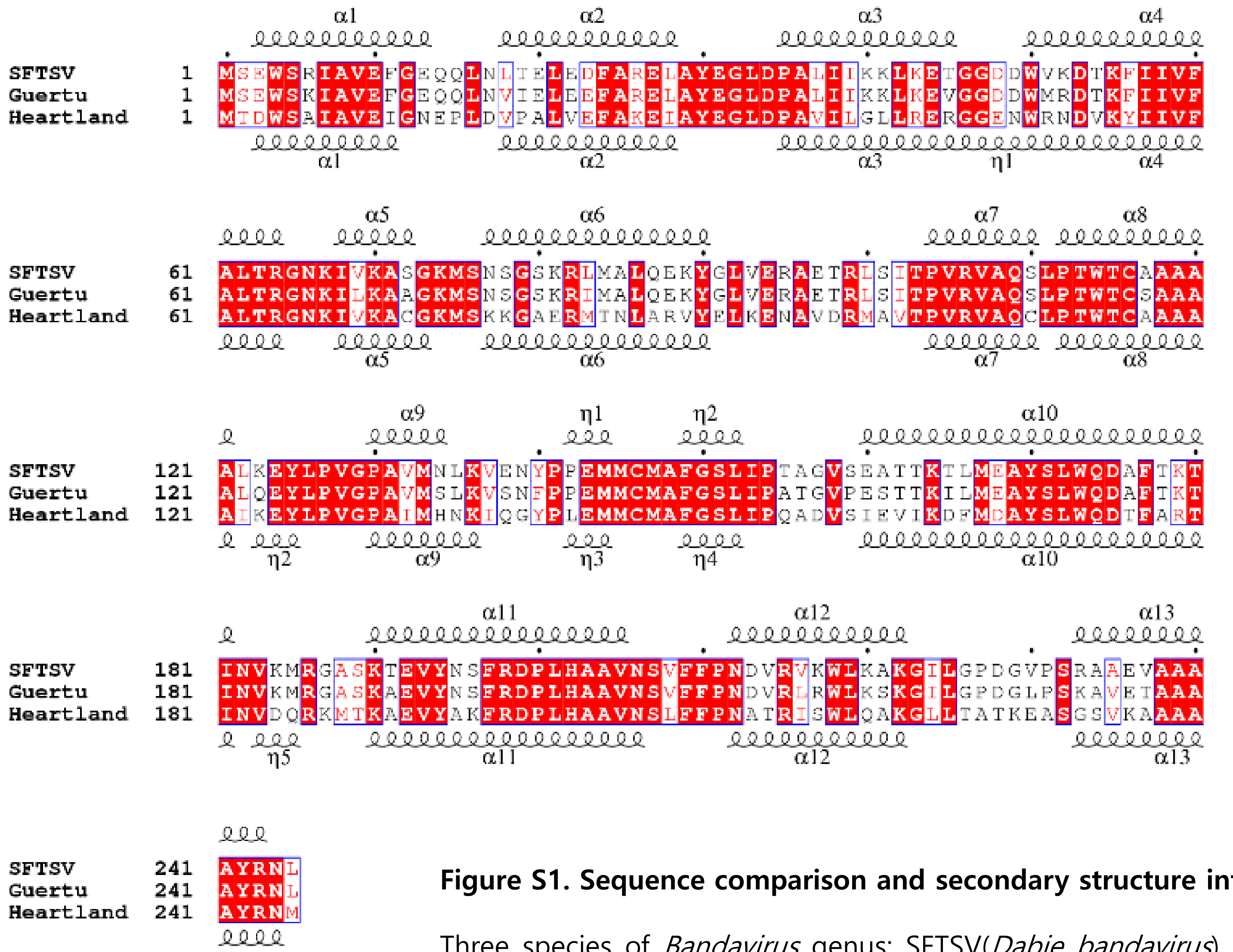

Figure S1. Sequence comparison and secondary structure information of *Bandavirus*.

Three species of *Bandavirus* genus; SFTSV(*Dabie bandavirus*), *Guertu bandavirus* and *Heartland bandavirus* were aligned and compared. Strictly conserved residues are boxed in white on a red background, and highly conserved residues are boxed in red on a white background. Every tenth residue is indicated with a dot. The secondary structure was work with a deposited SFTSV N structure in Protein Data Bank (PDB), and the α-helix is depicted by a coil (top : pentamer SFTSV N with PDB ID 4J4U, bottom : hexameric SFTSV N with PDB ID 4J4R). The figure was generated by ESPript3 (espript.ibcp.fr)

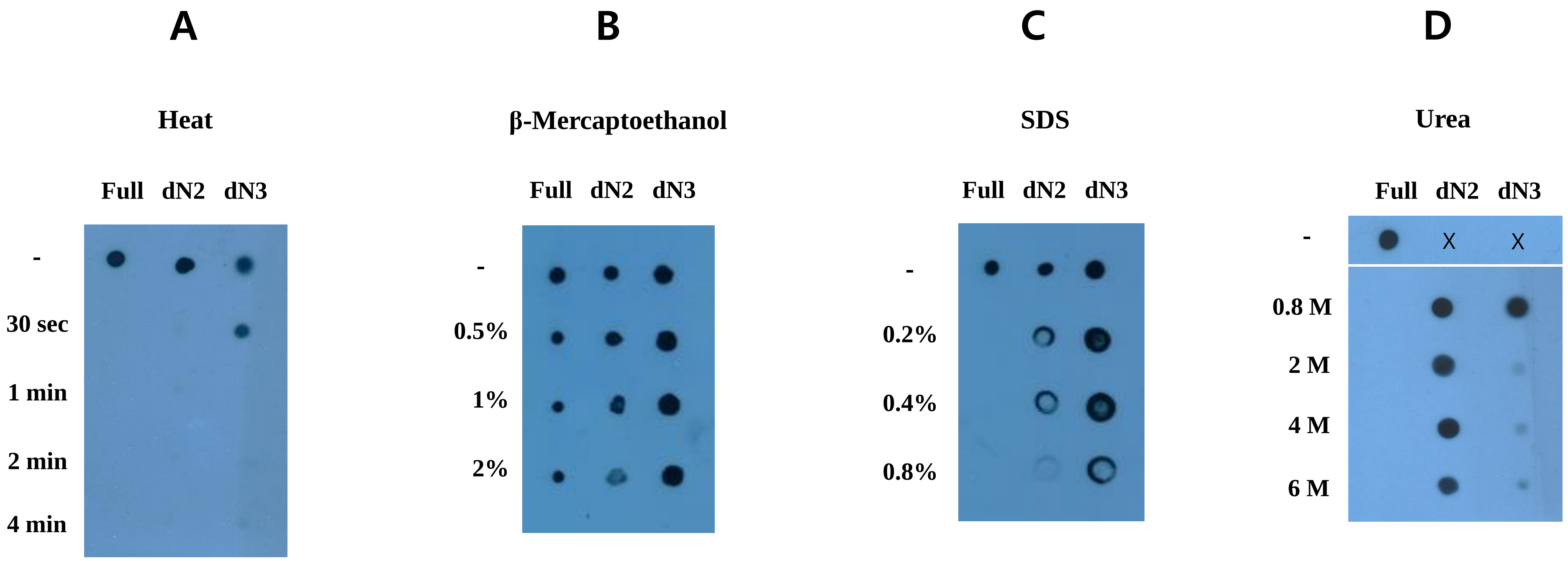

Figure S2. Epitope denaturation test.

Full-length and dN2 and dN3 of SFTSV N protein were tested with four denaturation factors; A: Urea, B: SDS, C: β-mercaptoethanol, D: Heat. SFTSV Ab clone #10 (B2H12) was used in Dot blot analysis. Under the urea and SDS conditions, the epitope of AB #10(B2H12) was denatured at full length, but it maintains the conformation at dN2 and dN3 (A,B). β-mercaptoethanol doesn't effect to the epitope at full length, as well as dN2 and dN3 (C). However, the epitope was denatured by heat treatment (D).

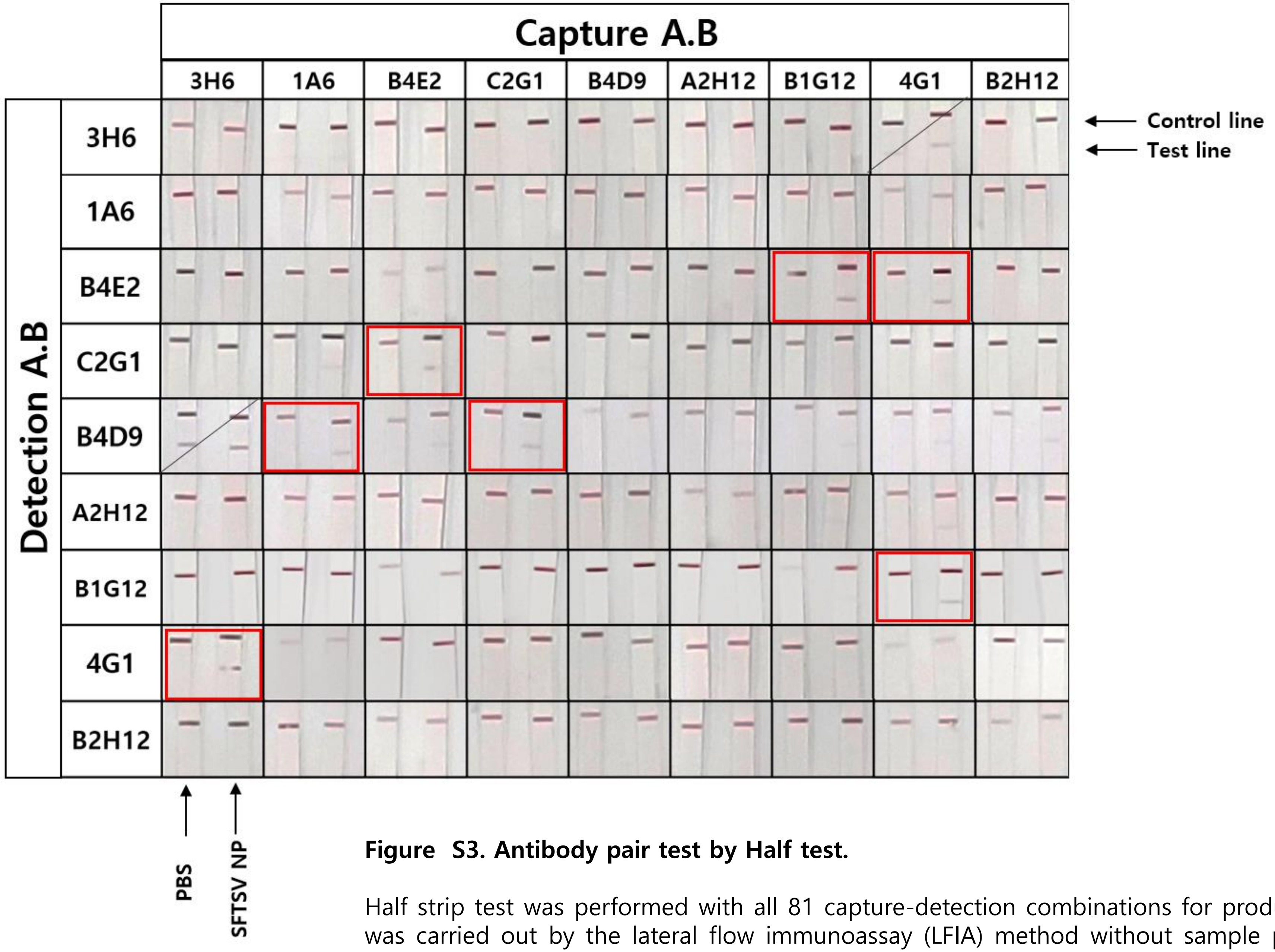

Figure S3. Antibody pair test by Half test.

Half strip test was performed with all 81 capture-detection combinations for produced 9 SFTSV N antibodies. It was carried out by the lateral flow immunoassay (LFIA) method without sample pad and conjugated pad. The test strip was coated with each of 9 SFTSV N antibodies as capture antibody, and dipped in the mixture of SFTSV N protein (100ng/ml) as antigen and each 9 antibodies as detection antibodies.
